# Supplementary material for: Northern Richness, Southern Dead End—Origin and Dispersal Events of Pseudolycoriella (Sciaridae, Diptera) between New Zealand’s Main Islands
Source: Insects. 2023 Jun 12;14(6):548. doi: 10.3390/insects14060548 (PMC10299329; doi:10.3390/insects14060548)
Supplement: Supplementary file 1 [file insects-14-00548-s001.zip › insects-2396243-supplementary.pdf]

Table S1. Sequenced specimens of *Pseudolycoriella* spp. (Sciaridae, Diptera) from New Zealand and Tasmania with GenBank accession numbers. The type of utilisation is indicated in the last column (H, Haplotype network construction; *B, *BEAST analyses). The collections of the voucher material are indicated (CBGC, Centre for Biodiversity Genomics, Guelph, Canada; NZAC, New Zealand Arthropod Collection, Auckland, New Zealand [collection number is given in brackets]; PABM, Private Collection Adam Broadley, Melbourne, Australia; SDEI, Senckenberg German Entomological Institute, Müncheberg, Germany).

Specimen number	Species	Taxonomic assignment	Voucher deposition	Island	Locality	Coordinates (lat./long.)	GenBank accession numbers			
							COI	16S	28S	
SDEI-Dipt-0000625	*Pseudolycoriella aotearoa*	*zealandica* clade	SDEI	North Island	Tongariro National Park	-39.393/175.450	OQ644691	OQ641704	-	
SDEI-Dipt-0000758	*Psl. aotearoa*	*zealandica* clade	SDEI	North Island	Tongariro National Park	-39.393/175.450	OQ569823	OQ581683	-	*B
SDEI-Dipt-0000978	*Psl. aotearoa*	*zealandica* clade	SDEI	North Island	Tongariro National Park	-39.393/175.450	OQ644701	-	-	
SDEI-Dipt-0000997	*Psl. aotearoa*	*zealandica* clade	NZAC (NZAC02028892)	North Island	Tongariro National Park	-39.393/175.450	MK906359	MK906451	MK906541	*B
SDEI-Dipt-0000999	*Psl. aotearoa*	*zealandica* clade	SDEI	North Island	Tongariro National Park	-39.393/175.450	OQ569853	OQ581708	-	*B
SDEI-Dipt-0001000	*Psl. aotearoa*	*zealandica* clade	NZAC (NZAC02029030)	North Island	Tongariro National Park	-39.393/175.450	OQ644702	OQ641707	-	
SDEI-Dipt-0001661	*Psl. aotearoa*	*zealandica* clade	SDEI	North Island	Tongariro National Park	-39.393/175.450	OQ644768	OQ641745	-	
SDEI-Dipt-0001662	*Psl. aotearoa*	*zealandica* clade	SDEI	North Island	Tongariro National Park	-39.393/175.450	OQ644769	OQ641746	-	
SDEI-Dipt-0001663	*Psl. aotearoa*	*zealandica* clade	SDEI	North Island	Tongariro National Park	-39.393/175.450	OQ644770	OQ641747	-	
SDEI-Dipt-0001665	*Psl. aotearoa*	*zealandica* clade	SDEI	North Island	Tongariro National Park	-39.393/175.450	OQ644771	OQ641749	-	
SDEI-Dipt-0001666	*Psl. aotearoa*	*zealandica* clade	SDEI	North Island	Tongariro National Park	-39.393/175.450	OQ573675	OQ598657	-	*B
SDEI-Dipt-0001667	*Psl. aotearoa*	*zealandica* clade	SDEI	North Island	Tongariro National Park	-39.393/175.450	OQ644772	OQ641750	-	
SDEI-Dipt-0001671	*Psl. aotearoa*	*zealandica* clade	SDEI	North Island	Tongariro National Park	-39.393/175.450	OQ644773	OQ641752	-	
SDEI-Dipt-0000844	*Psl. bispina*	*bispina* clade	SDEI	North Island	Pureora Forest Park	-38.5/175.583	OQ569829	OQ581689	-	*B
SDEI-Dipt-0000946	*Psl. bispina*	*bispina* clade	SDEI	North Island	Tongariro National Park	-39.376/175.444	MK906352	MK906444.2	MK906534	*B

Specimen number	Species	Taxonomic assignment	Voucher deposition	Island	Locality	Coordinates (lat./long.)	GenBank accession numbers			
							COI	16S	28S	
SDEI-Dipt-0000974	*Psl. bispina*	*bispina* clade	NZAC (NZAC02029036)	North Island	Pureora Forest Park	-38.5/175.583	MK906355	MK906447	MK906537	*B
SDEI-Dipt-0000975	*Psl. bispina*	*bispina* clade	NZAC (NZAC02029037)	North Island	Pureora Forest Park	-38.5/175.583	OQ644700	-	-	
SDEI-Dipt-0001251	*Psl. bispina*	*bispina* clade	SDEI	North Island	Katikati	-37.617/175.878	MK906384	MK906479.2	MK906569	*B
SDEI-Dipt-0001517	*Psl. bispina*	*bispina* clade	SDEI	North Island	Pureora Forest Park	-38.5/175.583	OQ573627	OQ598613	-	*B
SDEI-Dipt-0001518	*Psl. bispina*	*bispina* clade	SDEI	North Island	Pureora Forest Park	-38.5/175.583	MK906399	MK906497.2	MK906587	
SDEI-Dipt-0001522	*Psl. bispina*	*bispina* clade	NZAC (NZAC02029034)	North Island	Tongariro National Park	-39.376/175.444	MK906401	MK906499.2	MK906589	
SDEI-Dipt-0001526	*Psl. bispina*	*bispina* clade	NZAC (NZAC02029035)	North Island	Tongariro National Park	-39.376/175.444	OQ644738	-	-	
SDEI-Dipt-0001085	*Psl. cavatica*	*Psl. bruckii* group	NZAC (NZAC02029041)	North Island	Katikati	-37.566/175.895	MK906405	MK906455	MK906545	
SDEI-Dipt-0001297	*Psl. cavatica*	*Psl. bruckii* group	SDEI	North Island	Katikati	-37.538/175.930	OQ644717	OQ641715	-	
SDEI-Dipt-0001298	*Psl. cavatica*	*Psl. bruckii* group	NZAC (NZAC02029043)	North Island	Katikati	-37.538/175.930	OQ644718	OQ641716	-	
SDEI-Dipt-0001368	*Psl. cavatica*	*Psl. bruckii* group	SDEI	North Island	Katikati	-37.55/175.90	OQ644726	OQ641722	-	
SDEI-Dipt-0001377	*Psl. cavatica*	*Psl. bruckii* group	SDEI	North Island	Katikati	-37.542/175.920	OQ644727	OQ641723	-	
SDEI-Dipt-0001411	*Psl. cavatica*	*Psl. bruckii* group	SDEI	North Island	Katikati	-37.566/175.895	MK906395	MK906490	MK906580	
SDEI-Dipt-0001443	*Psl. cavatica*	*Psl. bruckii* group	SDEI	North Island	Katikati	-37.566/175.895	OQ644729	OQ641725	-	
SDEI-Dipt-0001458	*Psl. cavatica*	*Psl. bruckii* group	SDEI	North Island	Katikati	-37.566/175.895	OQ644730	OQ641726	-	
SDEI-Dipt-0000628	*Psl. dagae*	*jejuna* clade	SDEI	North Island	Katikati	-37.617/175.878	MK906330	MK906421	MK906511	
SDEI-Dipt-0000655	*Psl. dagae*	*jejuna* clade	SDEI	North Island	Katikati	-37.617/175.878	MK906331	MK906422	MK906512	*B
SDEI-Dipt-0000679	*Psl. dagae*	*jejuna* clade	NZAC (NZAC02028885)	North Island	Katikati	-37.617/175.878	OQ569820	OQ581682	-	*B

Specimen number	Species	Taxonomic assignment	Voucher deposition	Island	Locality	Coordinates (lat./long.)	GenBank accession numbers			
							COI	16S	28S	
SDEI-Dipt-0001249	*Psl. dagae*	*jejuna* clade	NZAC (NZAC02028898)	North Island	Katikati	-37.617/175.878	OQ644716	OQ641714	-	
SDEI-Dipt-0001367	*Psl. dagae*	*jejuna* clade	NZAC (NZAC02028899)	North Island	Katikati	-37.55/175.90	OQ644725	OQ641721	-	
SDEI-Dipt-0000568	*Psl. frederickedwardsi*	*macrotegmenta* s. str. clade	NZAC (NZAC02028903)	North Island	Pureora Forest	-38.5/175.583	OQ569816	-	-	*B
SDEI-Dipt-0000756	*Psl. frederickedwardsi*	*macrotegmenta* s. str. clade	SDEI	North Island	Tongariro National Park	-39.393/175.450	MK906339	MK906431.2	MK906521.2	
SDEI-Dipt-0000772	*Psl. frederickedwardsi*	*macrotegmenta* s. str. clade	SDEI	North Island	Tongariro National Park	-39.376/175.444	MK906341	MK906433	MK906523	*B
SDEI-Dipt-0000823	*Psl. frederickedwardsi*	*macrotegmenta* s. str. clade	SDEI	North Island	Pureora Forest Park	-38.5/175.583	OQ569828	OQ581688	-	*B
SDEI-Dipt-0000945	*Psl. frederickedwardsi*	*macrotegmenta* s. str. clade	NZAC (NZAC02028906)	North Island	Tongariro National Park	-39.376/175.444	OQ644697	-	-	
SDEI-Dipt-0000947	*Psl. frederickedwardsi*	*macrotegmenta* s. str. clade	NZAC (NZAC02028907)	North Island	Tongariro National Park	-39.376/175.444	OQ644698	-	-	
SDEI-Dipt-0001224	*Psl. frederickedwardsi*	*macrotegmenta* s. str. clade	NZAC (NZAC02028905)	North Island	Tongariro National Park	-39.393/175.450	MK906374	MK906468	MK906558	*B
SDEI-Dipt-0001237	*Psl. frederickedwardsi*	*macrotegmenta* s. str. clade	SDEI	North Island	Tongariro National Park	-39.393/175.450	MK906379	MK906474	MK906564	
SDEI-Dipt-0001247	*Psl. frederickedwardsi*	*macrotegmenta* s. str. clade	SDEI	North Island	Tongariro National Park	-39.393/175.450	MK906382	MK906477.2	MK906567	
SDEI-Dipt-0001507	*Psl. frederickedwardsi*	*macrotegmenta* s. str. clade	NZAC (NZAC02028902)	North Island	Pureora Forest	-38.5/175.583	OQ644732	-	-	
SDEI-Dipt-0001508	*Psl. frederickedwardsi*	*macrotegmenta* s. str. clade	SDEI	North Island	Pureora Forest	-38.5/175.583	OQ573625	OQ598611	-	*B
SDEI-Dipt-0001509	*Psl. frederickedwardsi*	*macrotegmenta* s. str. clade	SDEI	North Island	Pureora Forest	-38.5/175.583	OQ644733	-	-	
SDEI-Dipt-0001510	*Psl. frederickedwardsi*	*macrotegmenta* s. str. clade	SDEI	North Island	Pureora Forest	-38.5/175.583	OQ644734	-	-	
SDEI-Dipt-0001530	*Psl. frederickedwardsi*	*macrotegmenta* s. str. clade	NZAC (NZAC02028908)	North Island	Tongariro National Park	-39.376/175.444	OQ573630	OQ598616	-	*B
SDEI-Dipt-0001531	*Psl. frederickedwardsi*	*macrotegmenta* s. str. clade	SDEI	North Island	Tongariro National Park	-39.376/175.444	OQ644740	OQ641730	-	

Specimen number	Species	Taxonomic assignment	Voucher deposition	Island	Locality	Coordinates (lat./long.)	GenBank accession numbers			
							COI	16S	28S	
SDEI-Dipt-0001535	*Psl. frederickedwardsi*	*macrotegmenta* s. str. clade	SDEI	North Island	Tongariro National Park	-39.376/175.444	OQ644742	OQ641732	-	
SDEI-Dipt-0001537	*Psl. frederickedwardsi*	*macrotegmenta* s. str. clade	NZAC (NZAC02028904)	North Island	Tongariro National Park	-39.393/175.450	OQ573633	OQ598618	-	*B
SDEI-Dipt-0001603	*Psl. frederickedwardsi*	*macrotegmenta* s. str. clade	SDEI	North Island	Tongariro National Park	-39.393/175.450	OQ644744	OQ641733	-	
SDEI-Dipt-0001605	*Psl. frederickedwardsi*	*macrotegmenta* s. str. clade	SDEI	North Island	Tongariro National Park	-39.393/175.450	OQ644745	OQ641734	-	
SDEI-Dipt-0001606	*Psl. frederickedwardsi*	*macrotegmenta* s. str. clade	SDEI	North Island	Tongariro National Park	-39.393/175.450	OQ644746	OQ641735	-	
SDEI-Dipt-0001607	*Psl. frederickedwardsi*	*macrotegmenta* s. str. clade	SDEI	North Island	Tongariro National Park	-39.393/175.450	OQ644747	OQ641736	-	
SDEI-Dipt-0001608	*Psl. frederickedwardsi*	*macrotegmenta* s. str. clade	SDEI	North Island	Tongariro National Park	-39.393/175.450	OQ573671	OQ598655	-	*B
SDEI-Dipt-0001609	*Psl. frederickedwardsi*	*macrotegmenta* s. str. clade	SDEI	North Island	Tongariro National Park	-39.393/175.450	OQ644748	-	-	
SDEI-Dipt-0001610	*Psl. frederickedwardsi*	*macrotegmenta* s. str. clade	SDEI	North Island	Pureora Forest	-38.5/175.583	OQ573672	-	-	*B
SDEI-Dipt-0001611	*Psl. frederickedwardsi*	*macrotegmenta* s. str. clade	SDEI	North Island	Pureora Forest	-38.5/175.583	OQ644749	-	-	
SDEI-Dipt-0001612	*Psl. frederickedwardsi*	*macrotegmenta* s. str. clade	SDEI	North Island	Pureora Forest	-38.5/175.583	OQ644750	-	-	
SDEI-Dipt-0001614	*Psl. frederickedwardsi*	*macrotegmenta* s. str. clade	SDEI	North Island	Pureora Forest	-38.5/175.583	OQ644751	-	-	
SDEI-Dipt-0001616	*Psl. frederickedwardsi*	*macrotegmenta* s. str. clade	SDEI	North Island	Pureora Forest	-38.5/175.583	OQ644752	-	-	
SDEI-Dipt-0001620	*Psl. frederickedwardsi*	*macrotegmenta* s. str. clade	SDEI	North Island	Pureora Forest Park	-38.5/175.583	OQ644753	OQ641737	-	
SDEI-Dipt-0001621	*Psl. frederickedwardsi*	*macrotegmenta* s. str. clade	SDEI	North Island	Pureora Forest Park	-38.5/175.583	OQ644754	OQ641738	-	
SDEI-Dipt-0001655	*Psl. frederickedwardsi*	*macrotegmenta* s. str. clade	SDEI	North Island	Pureora Forest	-38.5/175.583	OQ644763	-	-	
SDEI-Dipt-0001656	*Psl. frederickedwardsi*	*macrotegmenta* s. str. clade	SDEI	North Island	Pureora Forest	-38.5/175.583	OQ644764	-	-	

Specimen number	Species	Taxonomic assignment	Voucher deposition	Island	Locality	Coordinates (lat./long.)	GenBank accession numbers			
							COI	16S	28S	
SDEI-Dipt-0001657	*Psl. frederickedwardsi*	*macrotegmenta* s. str. clade	SDEI	North Island	Pureora Forest	-38.5/175.583	OQ644765	-	-	
SDEI-Dipt-0001658	*Psl. frederickedwardsi*	*macrotegmenta* s. str. clade	SDEI	North Island	Pureora Forest	-38.5/175.583	OQ644766	-	-	
SDEI-Dipt-0001659	*Psl. frederickedwardsi*	*macrotegmenta* s. str. clade	SDEI	North Island	Pureora Forest	-38.5/175.583	OQ644767	-	-	
SDEI-Dipt-0000977	*Psl. gonotegmenta*	*macrotegmenta* s. str. clade	NZAC (NZAC02028912)	South Island	Maruia Springs	-42.382/172.262	MK906356	MK906448.2	-	*B
SDEI-Dipt-0000984	*Psl. gonotegmenta*	*macrotegmenta* s. str. clade	SDEI	South Island	Maruia Springs	-42.382/172.262	OQ569851	-	-	*B
SDEI-Dipt-0001495	*Psl. gonotegmenta*	*macrotegmenta* s. str. clade	NZAC (NZAC02028882)	South Island	Rahu Scenic Reserve	-42.273/171.980	OQ644731	OQ641727	MK906538	*B
SDEI-Dipt-0000506	*Psl. hauta*	*jejuna* clade	SDEI	South Island	Catlins	-46.541/169.608	MK906322	MK906412	MK906502.3	*B
SDEI-Dipt-0001134	*Psl. hauta*	*jejuna* clade	NZAC (NZAC02028878)	South Island	Catlins	-46.541/169.608	OQ644706	-	-	
SDEI-Dipt-0001137	*Psl. hauta*	*jejuna* clade	NZAC (NZAC02028913)	South Island	Catlins	-46.541/169.608	OQ644708	OQ641710	-	
SDEI-Dipt-0001142	*Psl. hauta*	*jejuna* clade	SDEI	South Island	Catlins	-46.541/169.608	OQ644709	OQ641711	-	
SDEI-Dipt-0000621	*Psl. huttoni*	*macrotegmenta* s. str. clade	NZAC (NZAC02028891)	North Island	Tongariro National Park	-39.393/175.450	MK906327	MK906418	MK906508	*B
SDEI-Dipt-0000892	*Psl. jaschhofi*	*macrotegmenta* s. l. clade	SDEI	South Island	Fiordland National Park	-44.686/167.964	OQ644696	OQ641706	-	
SDEI-Dipt-0000896	*Psl. jaschhofi*	*macrotegmenta* s. l. clade	SDEI	South Island	Fiordland National Park	-44.686/167.964	OQ569837	OQ581694	-	*B
SDEI-Dipt-0000962	*Psl. jaschhofi*	*macrotegmenta* s. l. clade	SDEI	South Island	Fiordland National Park	-45.000/168.010	OQ644699	-	-	
SDEI-Dipt-0001036	*Psl. jaschhofi*	*macrotegmenta* s. l. clade	NZAC (NZAC02028923)	South Island	Lewis Pass	-42.38/172.4	OQ644703	-	-	
SDEI-Dipt-0001198	*Psl. jaschhofi*	*macrotegmenta* s. l. clade	SDEI	South Island	Lewis Pass	-42.38/172.4	OQ644710	-	-	
SDEI-Dipt-0001199	*Psl. jaschhofi*	*macrotegmenta* s. l. clade	NZAC (NZAC02028889)	South Island	Lewis Pass	-42.38/172.4	OQ644711	-	-	

Specimen number	Species	Taxonomic assignment	Voucher deposition	Island	Locality	Coordinates (lat./long.)	GenBank accession numbers			
							COI	16S	28S	
SDEI-Dipt-0001203	*Psl. jaschhofi*	*macrotegmenta* s. l. clade	SDEI	South Island	Lewis Pass	-42.38/172.4	OQ644712	OQ641712	-	
SDEI-Dipt-0001207	*Psl. jaschhofi*	*macrotegmenta* s. l. clade	NZAC (NZAC02028929)	South Island	Lewis Pass	-42.38/172.4	OQ644713	-	-	
SDEI-Dipt-0001209	*Psl. jaschhofi*	*macrotegmenta* s. l. clade	NZAC (NZAC02028932)	South Island	Lewis Pass	-42.38/172.4	OQ573610	OQ598596	-	*B
SDEI-Dipt-0001210	*Psl. jaschhofi*	*macrotegmenta* s. l. clade	NZAC (NZAC02028931)	South Island	Lewis Pass	-42.38/172.4	OQ644714	OQ641713	-	
SDEI-Dipt-0001217	*Psl. jaschhofi*	*macrotegmenta* s. l. clade	SDEI	South Island	Lewis Pass	-42.38/172.4	OQ644715	-	-	
SDEI-Dipt-0001332	*Psl. jaschhofi*	*macrotegmenta* s. l. clade	NZAC (NZAC02028928)	South Island	Fiordland National Park	-44.686/167.964	OQ644719	OQ641717	-	
SDEI-Dipt-0001341	*Psl. jaschhofi*	*macrotegmenta* s. l. clade	NZAC (NZAC02028927)	South Island	Fiordland National Park	-44.686/167.964	OQ644720	OQ641718	-	
SDEI-Dipt-0001342	*Psl. jaschhofi*	*macrotegmenta* s. l. clade	NZAC (NZAC02028930)	South Island	Fiordland National Park	-44.686/167.964	OQ644721	-	-	
SDEI-Dipt-0001343	*Psl. jaschhofi*	*macrotegmenta* s. l. clade	NZAC (NZAC02028924)	South Island	Fiordland National Park	-44.699/168.108	OQ644722	OQ641719	-	
SDEI-Dipt-0001344	*Psl. jaschhofi*	*macrotegmenta* s. l. clade	SDEI	South Island	Fiordland National Park	-44.699/168.108	OQ644723	OQ641720	-	
SDEI-Dipt-0001345	*Psl. jaschhofi*	*macrotegmenta* s. l. clade	NZAC (NZAC02028925)	South Island	Fiordland National Park	-44.699/168.108	OQ644724	-	-	
SDEI-Dipt-0001511	*Psl. jaschhofi*	*macrotegmenta* s. l. clade	SDEI	South Island	Fiordland National Park	-44.699/168.108	MK906409	MK906494	MK906584	*B
SDEI-Dipt-0001512	*Psl. jaschhofi*	*macrotegmenta* s. l. clade	NZAC (NZAC02028933)	South Island	Fiordland National Park	-44.699/168.108	OQ644735	OQ641728	-	
SDEI-Dipt-0001513	*Psl. jaschhofi*	*macrotegmenta* s. l. clade	NZAC (NZAC02028926)	South Island	Fiordland National Park	-44.699/168.108	MK906410	MK906495	MK906585	
SDEI-Dipt-0001514	*Psl. jaschhofi*	*macrotegmenta* s. l. clade	SDEI	South Island	Fiordland National Park	-44.699/168.108	MK906398	MK906496	MK906586	
SDEI-Dipt-0001515	*Psl. jaschhofi*	*macrotegmenta* s. l. clade	SDEI	South Island	Fiordland National Park	-44.699/168.108	OQ573626	OQ598612	-	*B
SDEI-Dipt-0001643	*Psl. jaschhofi*	*macrotegmenta* s. l. clade	SDEI	South Island	Fiordland National Park	-44.699/168.108	OQ573673	OQ598656	-	*B

Specimen number	Species	Taxonomic assignment	Voucher deposition	Island	Locality	Coordinates (lat./long.)	GenBank accession numbers			
							COI	16S	28S	
SDEI-Dipt-0001644	*Psl. jaschhofi*	*macrotegmenta* s. l. clade	SDEI	South Island	Fiordland National Park	-44.699/168.108	OQ644755	OQ641739	-	
SDEI-Dipt-0001646	*Psl. jaschhofi*	*macrotegmenta* s. l. clade	SDEI	South Island	Fiordland National Park	-44.699/168.108	OQ644756	OQ641740	-	
SDEI-Dipt-0001648	*Psl. jaschhofi*	*macrotegmenta* s. l. clade	SDEI	South Island	Fiordland National Park	-44.699/168.108	OQ644757	OQ641741	-	
SDEI-Dipt-0001649	*Psl. jaschhofi*	*macrotegmenta* s. l. clade	SDEI	South Island	Fiordland National Park	-44.699/168.108	OQ644758	OQ641742	-	
SDEI-Dipt-0001650	*Psl. jaschhofi*	*macrotegmenta* s. l. clade	SDEI	South Island	Fiordland National Park	-44.699/168.108	OQ644759	OQ641743	-	
SDEI-Dipt-0001651	*Psl. jaschhofi*	*macrotegmenta* s. l. clade	SDEI	South Island	Fiordland National Park	-44.699/168.108	OQ644760	OQ641744	-	
SDEI-Dipt-0001652	*Psl. jaschhofi*	*macrotegmenta* s. l. clade	SDEI	South Island	Fiordland National Park	-44.699/168.108	OQ644761	-	-	
SDEI-Dipt-0001654	*Psl. jaschhofi*	*macrotegmenta* s. l. clade	SDEI	South Island	Fiordland National Park	-44.699/168.108	OQ644762	-	-	
SDEI-Dipt-0000579	*Psl. jejuna*	*jejuna* clade	SDEI	North Island	Pureora Forest	-38.5/175.583	OQ644689	-	-	
SDEI-Dipt-0000583	*Psl. jejuna*	*jejuna* clade	SDEI	North Island	Pureora Forest	-38.5/175.583	-	-	OQ640201	
SDEI-Dipt-0000588	*Psl. jejuna*	*jejuna* clade	SDEI	North Island	Pureora Forest	-38.5/175.583	OQ644690	-	-	
SDEI-Dipt-0000603	*Psl. jejuna*	*jejuna* clade	SDEI	North Island	Tongariro National Park	-39.393/175.450	MK906326	MK906416	MK906506	
SDEI-Dipt-0000617	*Psl. jejuna*	*jejuna* clade	SDEI	North Island	Tongariro National Park	-39.393/175.450	MK906403.2	MK906417.2	MK906507	*B
SDEI-Dipt-0000744	*Psl. jejuna*	*jejuna* clade	NZAC (NZAC02028937)	North Island	Tongariro National Park	-39.393/175.450	MK906404.2	MK906428.2	MK906518	
SDEI-Dipt-0000773	*Psl. jejuna*	*jejuna* clade	NZAC (NZAC02028936)	North Island	Tongariro National Park	-39.376/175.444	MK906342	MK906434.2	MK906524	*B
SDEI-Dipt-0000829	*Psl. jejuna*	*jejuna* clade	SDEI	North Island	Tongariro National Park	-39.376/175.444	MK906344	MK906436.2	MK906526.2	*B
SDEI-Dipt-0001193	*Psl. jejuna*	*jejuna* clade	SDEI	North Island	Tongariro National Park	-39.393/175.450	MK906371	MK906464.2	MK906554	

Specimen number	Species	Taxonomic assignment	Voucher deposition	Island	Locality	Coordinates (lat./long.)	GenBank accession numbers			
							COI	16S	28S	
SDEI-Dipt-0001195	*Psl. jejuna*	*jejuna* clade	NZAC (NZAC02028935)	North Island	Tongariro National Park	-39.393/175.450	MK906406	MK906466.2	MK906556	*B
SDEI-Dipt-0001339	*Psl. jejuna*	*jejuna* clade	SDEI	North Island	Tongariro National Park	-39.393/175.450	MK906390	MK906485	MK906575	*B
SDEI-Dipt-0001347	*Psl. jejuna*	*jejuna* clade	NZAC (NZAC02028934)	North Island	Tongariro National Park	-39.393/175.450	MK906391	MK906486.2	MK906576	*B
SDEI-Dipt-0000880	*Psl. jejunella*	*jejuna* clade	NZAC (NZAC02028876)	South Island	Fiordland National Park	-44.699/168.108	OQ644695	OQ641705	-	
SDEI-Dipt-0000935	*Psl. jejunella*	*jejuna* clade	SDEI	South Island	Fiordland National Park	-44.699/168.108	MK906351	MK906443	MK906533.2	*B
SDEI-Dipt-0000500	*Psl. kaikoura*	*bispina* clade	NZAC (NZAC02028890)	South Island	Waiau, Pillona	-42.656/173.041	MK906321	MK906411	MK906501	*B
SDEI-Dipt-0000615	*Psl. macrotegmenta*	*macrotegmenta* s. str. clade	SDEI	South Island	Ahaura, Granville State Forest	-42.351/171.542	OQ569818	-	-	*B
SDEI-Dipt-0000757	*Psl. macrotegmenta*	*macrotegmenta* s. str. clade	SDEI	North Island	Tongariro National Park	-39.393/175.450	MK906340	MK906432	MK906522	*B
SDEI-Dipt-0000859	*Psl. macrotegmenta*	*macrotegmenta* s. str. clade	SDEI	North Island	Tongariro National Park	-39.376/175.444	OQ644694	-	-	
SDEI-Dipt-0000627	*Psl. maddisoni*	*jejuna* clade	NZAC (NZAC02028883)	North Island	Katikati	-37.617/175.878	MK906329	MK906420	MK906510	
SDEI-Dipt-0000664	*Psl. maddisoni*	*jejuna* clade	SDEI	North Island	Katikati	-37.55/175.90	MK906332	MK906423	MK906513	*B
SDEI-Dipt-0001369	*Psl. maddisoni*	*jejuna* clade	SDEI	North Island	Katikati	-37.55/175.90	OQ573618	OQ598604	-	*B
SDEI-Dipt-0000595	*Psl. mahanga*	*bispina* clade	SDEI	South Island	Mt. Aspiring National Park	-44.231/169.231	MK906324	MK906414	MK906504	*B
SDEI-Dipt-0000904	*Psl. mahanga*	*bispina* clade	NZAC (NZAC02028948)	South Island	Fiordland National Park	-44.686/167.964	OQ569838	OQ581695	-	*B
SDEI-Dipt-0001008	*Psl. mahanga*	*bispina* clade	NZAC (NZAC02028884)	South Island	Fiordland National Park	-44.948/168.018	MK906362.2	MK906454	MK906544	
SDEI-Dipt-0000863	*Psl. orite*	*bispina* clade	NZAC (NZAC02028880)	North Island	Tongariro National Park	-39.376/175.444	MK906347	MK906439.2	MK906529.2	*B
SDEI-Dipt-0001519	*Psl. orite*	*bispina* clade	NZAC (NZAC02028952)	North Island	Tongariro National Park	-39.376/175.444	OQ573628	OQ598614	-	*B

Specimen number	Species	Taxonomic assignment	Voucher deposition	Island	Locality	Coordinates (lat./long.)	GenBank accession numbers			
							COI	16S	28S	
SDEI-Dipt-0001520	*Psl. orite*	*bispina* clade	SDEI	North Island	Tongariro National Park	-39.376/175.444	OQ644736	OQ641729	-	
SDEI-Dipt-0001521	*Psl. orite*	*bispina* clade	SDEI	North Island	Tongariro National Park	-39.376/175.444	MK906400	MK906498.2	MK906588	
SDEI-Dipt-0001525	*Psl. orite*	*bispina* clade	SDEI	North Island	Tongariro National Park	-39.376/175.444	OQ644737	-	-	
SDEI-Dipt-0001529	*Psl. orite*	*bispina* clade	NZAC (NZAC02028953)	North Island	Tongariro National Park	-39.376/175.444	OQ644739	-	-	
SDEI-Dipt-0001533	*Psl. orite*	*bispina* clade	SDEI	North Island	Tongariro National Park	-39.376/175.444	OQ644741	OQ641731	-	
SDEI-Dipt-0000560	*Psl. plicitegmenta*	*macrotegmenta* s. str. clade	NZAC (NZAC02028955)	South Island	Catlins	-46.541/169.608	OQ644688	-	-	
SDEI-Dipt-0000799	*Psl. plicitegmenta*	*macrotegmenta* s. str. clade	SDEI	South Island	Catlins	-46.466/169.525	OQ644692	-	-	
SDEI-Dipt-0001130	*Psl. plicitegmenta*	*macrotegmenta* s. str. clade	SDEI	South Island	Catlins	-46.541/169.608	OQ644704	OQ641708	-	
SDEI-Dipt-0001153	*Psl. plicitegmenta*	*macrotegmenta* s. str. clade	SDEI	South Island	Catlins	-46.541/169.608	MK906366	MK906459	MK906549	*B
SDEI-Dipt-0001155	*Psl. plicitegmenta*	*macrotegmenta* s. str. clade	NZAC (NZAC02028956)	South Island	Catlins	-46.541/169.608	OQ573595	OQ598581	-	*B
SDEI-Dipt-0001158	*Psl. plicitegmenta*	*macrotegmenta* s. str. clade	NZAC (NZAC02028879)	South Island	Catlins	-46.541/169.608	MK906367	MK906460	MK906550	
SDEI-Dipt-0001225	*Psl. porotaka*	*jejuna* clade	NZAC (NZAC02028887)	North Island	Katikati	-37.55/175.90	MK906375	MK906469	MK906559	*B
SDEI-Dipt-0001250	*Psl. porotaka*	*jejuna* clade	SDEI	North Island	Katikati	-37.617/175.878	MK906383	MK906478	MK906568	*B
SDEI-Dipt-0001005	*Psl. puhihi*	*bispina* clade	NZAC (NZAC02028958)	North Island	Katikati	-37.566/175.895	MK906361	MK906453	MK906543	*B
SDEI-Dipt-0001420	*Psl. puhihi*	*bispina* clade	SDEI	North Island	Katikati	-37.566/175.895	OQ573619	OQ598605	-	*B
SDEI-Dipt-0001422	*Psl. puhihi*	*bispina* clade	SDEI	North Island	Katikati	-37.566/175.895	MK906396	MK906491	MK906581	*B
SDEI-Dipt-0001523	*Psl. puhihi*	*bispina* clade	NZAC (NZAC02028823)	North Island	Tongariro National Park	-39.376/175.444	OQ573629	OQ598615	-	*B

Specimen number	Species	Taxonomic assignment	Voucher deposition	Island	Locality	Coordinates (lat./long.)	GenBank accession numbers			
							COI	16S	28S	
SDEI-Dipt-0000834	*Psl. raki*	*jejuna* clade	NZAC (NZAC02029013)	North Island	Waipoua Forest	-35.65/173.55	MK906345	MK906437	MK906527	*B
SDEI-Dipt-0001358	*Psl. robustotegmenta*	*macrotegmenta* s. str. clade	NZAC (NZAC02029012)	North Island	Katikati	-37.542/175.920	MK906392	MK906487.2	MK906577	*B
SDEI-Dipt-0001371	*Psl. robustotegmenta*	*macrotegmenta* s. str. clade	SDEI	North Island	Katikati	-37.55/175.90	MK906393	MK906488	MK906578	*B
GMNZA054-14	*Psl.* sp. I	*bispina* clade	CBGC	North Island	Waikato	-37.802/175.334	see [58]	-	-	*B
GMNZM463-14	*Psl.* sp. I	*bispina* clade	CBGC	North Island	Waikato	-37.802/175.334	see [58]	-	-	
GMNZU122-14	*Psl.* sp. I	*bispina* clade	CBGC	North Island	Waikato	-37.802/175.334	see [58]	-	-	
GMNZW091-14	*Psl.* sp. I	*bispina* clade	CBGC	North Island	Waikato	-37.802/175.334	see [58]	-	-	*B
SDEI-Dipt-0000745	*Psl.* sp. II	*zealandica* clade	SDEI	South Island	Cass	-43.033/171.75	MK906337	MK906429	MK906519	*B
SDEI-Dipt-0001740	*Psl.* sp. TAS-1	*macrotegmenta* s. l. clade	SDEI (temporarily)	Tasmania	Mt. Weld	-43.070/146.670	OQ644774	OQ641754	-	
SDEI-Dipt-0001751	*Psl.* sp. TAS-1	*macrotegmenta* s. l. clade	SDEI (temporarily)	Tasmania	Mt. Weld	-43.070/146.670	OQ573681	OQ598661	OQ606886	*B
SDEI-Dipt-0001747	*Psl.* sp. TAS-2	*macrotegmenta* s. l. clade	SDEI (temporarily)	Tasmania	Mt. Weld	-43.070/146.670	OQ573679	OQ598659	OQ606885	*B
SDEI-Dipt-0001750	*Psl.* sp. TAS-2	*macrotegmenta* s. l. clade	SDEI (temporarily)	Tasmania	Mt. Weld	-43.070/146.670	OQ573680	OQ598660	-	*B
SDEI-Dipt-0001742	*Psl.* sp. TAS-3	*macrotegmenta* s. str. clade	SDEI (temporarily)	Tasmania	Mt. Weld	-43.070/146.670	OQ573678	OQ598658	OQ606884	*B
SDEI-Dipt-0001133	*Psl. subtilitegmenta*	*macrotegmenta* s. l. clade	SDEI	South Island	Catlins	-46.541/169.608	OQ644705	OQ641709	-	
SDEI-Dipt-0001136	*Psl. subtilitegmenta*	*macrotegmenta* s. l. clade	NZAC (NZAC02028961)	South Island	Catlins	-46.541/169.608	OQ644707	-	-	
SDEI-Dipt-0001151	*Psl. subtilitegmenta*	*macrotegmenta* s. l. clade	NZAC (NZAC02028893)	South Island	Catlins	-46.541/169.608	MK906364	MK906457	MK906547	*B
SDEI-Dipt-0000885	*Psl. sudhausi*	*jejuna* clade	NZAC (NZAC02028814)	South Island	Fiordland National Park	-44.699/168.108	MK906348	MK906440	MK906530	*B/H
SDEI-Dipt-0000922	*Psl. sudhausi*	*jejuna* clade	NZAC (NZAC02028822)	South Island	Fiordland National Park	-44.699/168.108	OQ569840	OQ581697	-	H

Specimen number	Species	Taxonomic assignment	Voucher deposition	Island	Locality	Coordinates (lat./long.)	GenBank accession numbers			
							COI	16S	28S	
SDEI-Dipt-0000929	*Psl. sudhausi*	*jejuna* clade	SDEI	South Island	Fiordland National Park	-44.699/168.108	OQ569844	OQ581701	-	H
SDEI-Dipt-0001009	*Psl. sudhausi*	*jejuna* clade	NZAC (NZAC02028821)	South Island	Fiordland National Park	-44.948/168.018	OQ569857	OQ598565	-	H
SDEI-Dipt-0001011	*Psl. sudhausi*	*jejuna* clade	SDEI	South Island	Fiordland National Park	-44.948/168.018	OQ569858	-	-	H
SDEI-Dipt-0001013	*Psl. sudhausi*	*jejuna* clade	NZAC (NZAC02028819)	South Island	Fiordland National Park	-44.948/168.018	OQ569860	-	-	H
SDEI-Dipt-0001071	*Psl. sudhausi*	*jejuna* clade	SDEI	Stewart Island	Stewart Island	-46.890/168.022	OQ569868	OQ598572	-	*B/H
SDEI-Dipt-0001090	*Psl. sudhausi*	*jejuna* clade	NZAC (NZAC02028894)	Stewart Island	Stewart Island	-46.898/168.073	OQ569869	OQ598573	OQ606882	*B/H
SDEI-Dipt-0001093	*Psl. sudhausi*	*jejuna* clade	SDEI	Stewart Island	Stewart Island	-46.898/168.073	OQ573586	OQ598574	-	*B/H
SDEI-Dipt-0001116	*Psl. sudhausi*	*jejuna* clade	SDEI	Stewart Island	Stewart Island	-46.890/168.022	OQ573587	OQ598575	-	H
SDEI-Dipt-0001146	*Psl. sudhausi*	*jejuna* clade	SDEI	South Island	Fiordland National Park	-44.923/168.041	OQ573591	OQ598578	-	H
SDEI-Dipt-0001147	*Psl. sudhausi*	*jejuna* clade	NZAC (NZAC02028815)	South Island	Fiordland National Park	-44.923/168.041	OQ573592	OQ598579	-	H
SDEI-Dipt-0001148	*Psl. sudhausi*	*jejuna* clade	NZAC (NZAC02028816)	South Island	Fiordland National Park	-44.923/168.041	OQ573593	OQ598580	-	H
SDEI-Dipt-0001149	*Psl. sudhausi*	*jejuna* clade	SDEI	South Island	Fiordland National Park	-44.923/168.041	OQ573594	-	-	H
SDEI-Dipt-0001167	*Psl. sudhausi*	*jejuna* clade	NZAC (NZAC02028818)	South Island	Fiordland National Park	-44.906/168.063	MK906368	MK906461.2	MK906551	H
SDEI-Dipt-0001170	*Psl. sudhausi*	*jejuna* clade	SDEI	South Island	Fiordland National Park	-44.906/168.063	MK906369	MK906462	MK906552	H
SDEI-Dipt-0001175	*Psl. sudhausi*	*jejuna* clade	NZAC (NZAC02028817)	South Island	Fiordland National Park	-44.906/168.063	MK906370	MK906463.2	MK906553	*B/H
SDEI-Dipt-0001177	*Psl. sudhausi*	*jejuna* clade	SDEI	South Island	Fiordland National Park	-44.923/168.041	OQ573604	OQ598590	-	*B/H
SDEI-Dipt-0001459	*Psl. sudhausi*	*jejuna* clade	SDEI	South Island	Fiordland National Park	-44.906/168.063	MK906397	MK906493.2	MK906583	H

Specimen number	Species	Taxonomic assignment	Voucher deposition	Island	Locality	Coordinates (lat./long.)	GenBank accession numbers			
							COI	16S	28S	
SDEI-Dipt-0001528	*Psl. teo*	*bispina* clade	NZAC (NZAC02028895)	North Island	Tongariro National Park	-39.376/175.444	MK906402	MK906500.2	MK906590	*B
SDEI-Dipt-0000513	*Psl. tewaipounamu*	*jejuna* clade	NZAC (NZAC02028861)	South Island	Mt. Aspiring National Park	-44.231/169.231	MK906323	MK906413	MK906503	H
SDEI-Dipt-0000614	*Psl. tewaipounamu*	*jejuna* clade	NZAC (NZAC02028977)	South Island	Catlins	-46.466/169.525	OQ569817	OQ581680	-	H
SDEI-Dipt-0000752	*Psl. tewaipounamu*	*jejuna* clade	SDEI	South Island	Maruia Forest	-42.033/172.293	MK906338	MK906430.2	MK906520	*B/H
SDEI-Dipt-0000788	*Psl. tewaipounamu*	*jejuna* clade	NZAC (NZAC02028871)	South Island	Maruia Forest	-42.033/172.293	OQ569825	OQ581685	-	H
SDEI-Dipt-0000801	*Psl. tewaipounamu*	*jejuna* clade	NZAC (NZAC02028976)	South Island	Catlins	-46.466/169.525	OQ569826	OQ581686	-	*B/H
SDEI-Dipt-0000814	*Psl. tewaipounamu*	*jejuna* clade	SDEI	South Island	Catlins	-46.466/169.525	OQ644693	OQ581687	-	H
SDEI-Dipt-0000871	*Psl. tewaipounamu*	*jejuna* clade	SDEI	South Island	Fiordland National Park	-44.913/168.047	OQ569831	-	-	H
SDEI-Dipt-0000879	*Psl. tewaipounamu*	*jejuna* clade	SDEI	South Island	Fiordland National Park	-44.699/168.108	OQ569832	OQ581690	-	H
SDEI-Dipt-0000881	*Psl. tewaipounamu*	*jejuna* clade	SDEI	South Island	Fiordland National Park	-44.699/168.108	OQ569833	OQ581691	-	H
SDEI-Dipt-0000907	*Psl. tewaipounamu*	*jejuna* clade	SDEI	South Island	Fiordland National Park	-44.699/168.108	MK906350	MK906442	MK906532.2	H
SDEI-Dipt-0000910	*Psl. tewaipounamu*	*jejuna* clade	NZAC (NZAC02028862)	South Island	Fiordland National Park	-44.699/168.108	OQ569839	OQ581696	-	H
SDEI-Dipt-0000924	*Psl. tewaipounamu*	*jejuna* clade	SDEI	South Island	Fiordland National Park	-44.699/168.108	OQ569841	OQ581698	-	H
SDEI-Dipt-0000927	*Psl. tewaipounamu*	*jejuna* clade	NZAC (NZAC02028863)	South Island	Fiordland National Park	-44.699/168.108	OQ569842	OQ581699	-	H
SDEI-Dipt-0000928	*Psl. tewaipounamu*	*jejuna* clade	NZAC (NZAC02028864)	South Island	Fiordland National Park	-44.699/168.108	OQ569843	OQ581700	-	H
SDEI-Dipt-0000934	*Psl. tewaipounamu*	*jejuna* clade	NZAC (NZAC02028858)	South Island	Fiordland National Park	-44.699/168.108	OQ569845	OQ581702	-	H
SDEI-Dipt-0000942	*Psl. tewaipounamu*	*jejuna* clade	SDEI	South Island	Maruia Springs	-42.382/172.262	OQ569846	-	-	H

Specimen number	Species	Taxonomic assignment	Voucher deposition	Island	Locality	Coordinates (lat./long.)	GenBank accession numbers			
							COI	16S	28S	
SDEI-Dipt-0000950	*Psl. tewaipounamu*	*jejuna* clade	SDEI	South Island	Fiordland National Park	-45.000/168.010	OQ569847	OQ581703	-	H
SDEI-Dipt-0000952	*Psl. tewaipounamu*	*jejuna* clade	NZAC (NZAC02028852)	South Island	Fiordland National Park	-45.000/168.010	OQ569848	OQ581704	-	H
SDEI-Dipt-0000956	*Psl. tewaipounamu*	*jejuna* clade	NZAC (NZAC02028896)	South Island	Fiordland National Park	-45.000/168.010	MK906353	MK906445	MK906535	*B/H
SDEI-Dipt-0000960	*Psl. tewaipounamu*	*jejuna* clade	NZAC (NZAC02028867)	South Island	Maruia Forest	-42.033/172.293	MK906354.2	MK906446	MK906536	H
SDEI-Dipt-0000964	*Psl. tewaipounamu*	*jejuna* clade	SDEI	South Island	Fiordland National Park	-45.000/168.010	OQ569849	OQ581705	-	H
SDEI-Dipt-0000973	*Psl. tewaipounamu*	*jejuna* clade	SDEI	South Island	Maruia Springs	-42.382/172.262	OQ569850	OQ581706	-	*B/H
SDEI-Dipt-0000988	*Psl. tewaipounamu*	*jejuna* clade	NZAC (NZAC02028869)	South Island	Maruia Springs	-42.382/172.262	OQ569852	OQ581707	-	H
SDEI-Dipt-0000994	*Psl. tewaipounamu*	*jejuna* clade	SDEI	South Island	Fiordland National Park	-44.808/168.109	MK906358	MK906450	MK906540.2	H
SDEI-Dipt-0001006	*Psl. tewaipounamu*	*jejuna* clade	NZAC (NZAC02028855)	South Island	Fiordland National Park	-44.948/168.018	OQ569856	OQ598564	-	H
SDEI-Dipt-0001012	*Psl. tewaipounamu*	*jejuna* clade	SDEI	South Island	Fiordland National Park	-44.948/168.018	OQ569859	-	-	H
SDEI-Dipt-0001014	*Psl. tewaipounamu*	*jejuna* clade	NZAC (NZAC02028856)	South Island	Fiordland National Park	-44.948/168.018	OQ569861	-	-	H
SDEI-Dipt-0001016	*Psl. tewaipounamu*	*jejuna* clade	NZAC (NZAC02028835)	South Island	Fiordland National Park	-44.948/168.018	OQ569862	OQ598566	-	H
SDEI-Dipt-0001017	*Psl. tewaipounamu*	*jejuna* clade	SDEI	South Island	Fiordland National Park	-44.948/168.018	OQ569863	OQ598567	-	H
SDEI-Dipt-0001025	*Psl. tewaipounamu*	*jejuna* clade	SDEI	South Island	Fiordland National Park	-44.948/168.018	OQ569864	OQ598568	-	H
SDEI-Dipt-0001038	*Psl. tewaipounamu*	*jejuna* clade	SDEI	South Island	Maruia Forest	-42.033/172.293	OQ569865	OQ598569	-	H
SDEI-Dipt-0001044	*Psl. tewaipounamu*	*jejuna* clade	SDEI	South Island	Maruia Forest	-42.033/172.293	OQ569866	OQ598570	-	H
SDEI-Dipt-0001045	*Psl. tewaipounamu*	*jejuna* clade	NZAC (NZAC02028868)	South Island	Maruia Forest	-42.033/172.293	OQ569867	OQ598571	-	*B/H
SDEI-Dipt-0001132	*Psl. tewaipounamu*	*jejuna* clade	SDEI	South Island	Catlins	-46.541/169.608	OQ573588	OQ598576	OQ606883	*B/H

Specimen number	Species	Taxonomic assignment	Voucher deposition	Island	Locality	Coordinates (lat./long.)	GenBank accession numbers			
							COI	16S	28S	
SDEI-Dipt-0001139	*Psl. tewaipounamu*	*jejuna* clade	NZAC (NZAC02028838)	South Island	Catlins	-46.541/169.608	OQ573589	OQ598577	OQ640203	*B/H
SDEI-Dipt-0001143	*Psl. tewaipounamu*	*jejuna* clade	SDEI	South Island	Fiordland National Park	-44.923/168.041	OQ573590	-	-	H
SDEI-Dipt-0001156	*Psl. tewaipounamu*	*jejuna* clade	SDEI	South Island	Catlins	-46.541/169.608	OQ573596	OQ598582	OQ640202	*B/H
SDEI-Dipt-0001165	*Psl. tewaipounamu*	*jejuna* clade	SDEI	South Island	Fiordland National Park	-44.906/168.063	OQ573599	OQ598585	-	H
SDEI-Dipt-0001169	*Psl. tewaipounamu*	*jejuna* clade	NZAC (NZAC02028854)	South Island	Fiordland National Park	-44.906/168.063	OQ573600	OQ598586	-	H
SDEI-Dipt-0001172	*Psl. tewaipounamu*	*jejuna* clade	SDEI	South Island	Fiordland National Park	-44.906/168.063	OQ573601	OQ598587	-	H
SDEI-Dipt-0001173	*Psl. tewaipounamu*	*jejuna* clade	NZAC (NZAC02028853)	South Island	Fiordland National Park	-44.906/168.063	OQ573602	OQ598588	-	H
SDEI-Dipt-0001174	*Psl. tewaipounamu*	*jejuna* clade	SDEI	South Island	Fiordland National Park	-44.906/168.063	OQ573603	OQ598589	-	H
SDEI-Dipt-0001179	*Psl. tewaipounamu*	*jejuna* clade	NZAC (NZAC02028866)	South Island	Maruia Forest	-42.033/172.293	OQ573605	OQ598591	-	H
SDEI-Dipt-0001180	*Psl. tewaipounamu*	*jejuna* clade	SDEI	South Island	Maruia Forest	-42.033/172.293	OQ573606	OQ598592	-	H
SDEI-Dipt-0001181	*Psl. tewaipounamu*	*jejuna* clade	SDEI	South Island	Maruia Forest	-42.033/172.293	OQ573607	OQ598593	-	H
SDEI-Dipt-0001206	*Psl. tewaipounamu*	*jejuna* clade	NZAC (NZAC02028857)	South Island	Fiordland National Park	-44.923/168.041	OQ573609	OQ598595	-	*B/H
SDEI-Dipt-0001481	*Psl. tewaipounamu*	*jejuna* clade	NZAC (NZAC02028860)	South Island	Rahu Scenic Reserve	-42.273/171.980	OQ573620	OQ598606	-	H
SDEI-Dipt-0001490	*Psl. tewaipounamu*	*jejuna* clade	NZAC (NZAC02028859)	South Island	Rahu Scenic Reserve	-42.273/171.980	OQ573621	OQ598607	-	*B/H
SDEI-Dipt-0001492	*Psl. tewaipounamu*	*jejuna* clade	SDEI	South Island	Rahu Scenic Reserve	-42.273/171.980	OQ573622	OQ598608	-	H
SDEI-Dipt-0001493	*Psl. tewaipounamu*	*jejuna* clade	SDEI	South Island	Rahu Scenic Reserve	-42.273/171.980	OQ573623	OQ598609	-	H
SDEI-Dipt-0001506	*Psl. tewaipounamu*	*jejuna* clade	SDEI	South Island	Mt. Aspiring National Park	-44.231/169.231	OQ573624	OQ598610	-	H
SDEI-Dipt-0000531	*Psl. tonnoiri*	*macrotegmenta* s. str. clade	NZAC (NZAC02028979)	South Island	Catlins	-46.541/169.608	OQ569815	-	-	H

Specimen number	Species	Taxonomic assignment	Voucher deposition	Island	Locality	Coordinates (lat./long.)	GenBank accession numbers			
							COI	16S	28S	
SDEI-Dipt-0000597	*Psl. tonnoiri*	*macrotegmenta* s. str. clade	SDEI	North Island	Tongariro National Park	-39.393/175.450	MK906325	MK906415	MK906505	H
SDEI-Dipt-0000719	*Psl. tonnoiri*	*macrotegmenta* s. str. clade	NZAC (NZAC02028980)	South Island	Cass	-43.033/171.75	OQ569821	-	-	H
SDEI-Dipt-0000722	*Psl. tonnoiri*	*macrotegmenta* s. str. clade	SDEI	South Island	Cass	-43.033/171.75	OQ569822	-	-	H
SDEI-Dipt-0000813	*Psl. tonnoiri*	*macrotegmenta* s. str. clade	NZAC (NZAC02028981)	North Island	Tongariro National Park	-39.376/175.444	OQ569827	-	-	H
SDEI-Dipt-0000856	*Psl. tonnoiri*	*macrotegmenta* s. str. clade	NZAC (NZAC02028872)	North Island	Tongariro National Park	-39.376/175.444	OQ569830	-	-	H
SDEI-Dipt-0000980	*Psl. tonnoiri*	*macrotegmenta* s. str. clade	SDEI	North Island	Tongariro National Park	-39.393/175.450	MK906357	MK906449	MK906539	*B/H
SDEI-Dipt-0001140	*Psl. tonnoiri*	*macrotegmenta* s. str. clade	SDEI	South Island	Catlins	-46.541/169.608	MK906363	MK906456	MK906546	H
SDEI-Dipt-0001152	*Psl. tonnoiri*	*macrotegmenta* s. str. clade	SDEI	South Island	Catlins	-46.541/169.608	MK906365	MK906458	MK906548	*B/H
SDEI-Dipt-0001532	*Psl. tonnoiri*	*macrotegmenta* s. str. clade	NZAC (NZAC02028982)	North Island	Tongariro National Park	-39.376/175.444	OQ573631	OQ598617	-	*B/H
SDEI-Dipt-0001534	*Psl. tonnoiri*	*macrotegmenta* s. str. clade	SDEI	North Island	Tongariro National Park	-39.376/175.444	OQ573632	-	-	H
SDEI-Dipt-0001536	*Psl. tonnoiri*	*macrotegmenta* s. str. clade	NZAC (NZAC02028978)	North Island	Tongariro National Park	-39.393/175.450	OQ644743	-	-	
SDEI-Dipt-0001588	*Psl. tonnoiri*	*macrotegmenta* s. str. clade	SDEI	North Island	Tongariro National Park	-39.376/175.444	OQ573656	OQ598640	-	H
SDEI-Dipt-0001590	*Psl. tonnoiri*	*macrotegmenta* s. str. clade	SDEI	North Island	Tongariro National Park	-39.393/175.450	OQ573657	OQ598641	-	H
SDEI-Dipt-0001591	*Psl. tonnoiri*	*macrotegmenta* s. str. clade	SDEI	North Island	Tongariro National Park	-39.393/175.450	OQ573658	OQ598642	-	H
SDEI-Dipt-0001592	*Psl. tonnoiri*	*macrotegmenta* s. str. clade	SDEI	North Island	Tongariro National Park	-39.393/175.450	OQ573659	OQ598643	-	H
SDEI-Dipt-0001593	*Psl. tonnoiri*	*macrotegmenta* s. str. clade	SDEI	North Island	Tongariro National Park	-39.393/175.450	OQ573660	OQ598644	-	H
SDEI-Dipt-0001594	*Psl. tonnoiri*	*macrotegmenta* s. str. clade	SDEI	North Island	Tongariro National Park	-39.393/175.450	OQ573661	OQ598645	-	H

Specimen number	Species	Taxonomic assignment	Voucher deposition	Island	Locality	Coordinates (lat./long.)	GenBank accession numbers			
							COI	16S	28S	
SDEI-Dipt-0001595	*Psl. tonnoiri*	*macrotegmenta* s. str. clade	SDEI	North Island	Tongariro National Park	-39.393/175.450	OQ573662	OQ598646	-	H
SDEI-Dipt-0001596	*Psl. tonnoiri*	*macrotegmenta* s. str. clade	SDEI	North Island	Tongariro National Park	-39.393/175.450	OQ573663	OQ598647	-	H
SDEI-Dipt-0001597	*Psl. tonnoiri*	*macrotegmenta* s. str. clade	PABM	North Island	Tongariro National Park	-39.393/175.450	OQ573664	OQ598648	-	H
SDEI-Dipt-0001598	*Psl. tonnoiri*	*macrotegmenta* s. str. clade	SDEI	North Island	Tongariro National Park	-39.393/175.450	OQ573665	OQ598649	-	H
SDEI-Dipt-0001599	*Psl. tonnoiri*	*macrotegmenta* s. str. clade	SDEI	North Island	Tongariro National Park	-39.393/175.450	OQ573666	OQ598650	-	H
SDEI-Dipt-0001600	*Psl. tonnoiri*	*macrotegmenta* s. str. clade	SDEI	North Island	Tongariro National Park	-39.393/175.450	OQ573667	OQ598651	-	H
SDEI-Dipt-0001601	*Psl. tonnoiri*	*macrotegmenta* s. str. clade	SDEI	North Island	Tongariro National Park	-39.393/175.450	OQ573668	OQ598652	-	H
SDEI-Dipt-0001602	*Psl. tonnoiri*	*macrotegmenta* s. str. clade	SDEI	North Island	Tongariro National Park	-39.393/175.450	OQ573669	OQ598653	-	H
SDEI-Dipt-0001604	*Psl. tonnoiri*	*macrotegmenta* s. str. clade	SDEI	North Island	Tongariro National Park	-39.393/175.450	OQ573670	OQ598654	-	H
SDEI-Dipt-0001194	*Psl. tuakana*	*jejuna* clade	NZAC (NZAC02028897)	North Island	Tongariro National Park	-39.393/175.450	MK906372	MK906465.2	MK906555	*B
SDEI-Dipt-0001337	*Psl. tuakana*	*jejuna* clade	SDEI	North Island	Tongariro National Park	-39.393/175.450	MK906389	MK906484.2	MK906574	
SDEI-Dipt-0000665	*Psl. wernermohrigi*	*macrotegmenta* s. str. clade	SDEI	North Island	Katikati	-37.55/175.90	MK906333	MK906424	MK906514	*B
SDEI-Dipt-0000670	*Psl. wernermohrigi*	*macrotegmenta* s. str. clade	NZAC (NZAC02028873)	North Island	Katikati	-37.55/175.90	MK906334	MK906425	MK906515	
SDEI-Dipt-0000674	*Psl. wernermohrigi*	*macrotegmenta* s. str. clade	NZAC (NZAC02028874)	North Island	Katikati	-37.55/175.90	MK906335	MK906426	MK906516	*B
SDEI-Dipt-0000681	*Psl.* cf. *wernermohrigi*	*macrotegmenta* s. str. clade	SDEI	North Island	Katikati	-37.55/175.90	MK906336	MK906427	MK906517	*B
SDEI-Dipt-0001223	*Psl. wernermohrigi*	*macrotegmenta* s. str. clade	NZAC (NZAC02029047)	North Island	Katikati	-37.55/175.90	MK906373	MK906467	MK906557	
SDEI-Dipt-0001227	*Psl. wernermohrigi*	*macrotegmenta* s. str. clade	NZAC (NZAC02028987)	North Island	Katikati	-37.55/175.90	MK906376	MK906470	MK906560	

Specimen number	Species	Taxonomic assignment	Voucher deposition	Island	Locality	Coordinates (lat./long.)	GenBank accession numbers COI	16S	28S	
SDEI-Dipt-0001232	*Psl.* cf. *wernermohrigi*	*macrotegmenta* s. str. clade	SDEI	North Island	Katikati	-37.55/175.90	MK906377	MK906471	MK906561	
SDEI-Dipt-0001234	*Psl. wernermohrigi*	*macrotegmenta* s. str. clade	SDEI	North Island	Katikati	-37.55/175.90	MK906378	MK906472	MK906562	
SDEI-Dipt-0001235	*Psl. wernermohrigi*	*macrotegmenta* s. str. clade	SDEI	North Island	Katikati	-37.55/175.90	MK906407	MK906473	MK906563	*B
SDEI-Dipt-0001238	*Psl. wernermohrigi*	*macrotegmenta* s. str. clade	NZAC (NZAC02028983)	North Island	Katikati	-37.55/175.90	MK906380	MK906475	MK906565	
SDEI-Dipt-0001245	*Psl. wernermohrigi*	*macrotegmenta* s. str. clade	NZAC (NZAC02028986)	North Island	Katikati	-37.55/175.90	MK906381	MK906476	MK906566	
SDEI-Dipt-0001270	*Psl. wernermohrigi*	*macrotegmenta* s. str. clade	NZAC (NZAC02028985)	North Island	Pukororo Miranda	-37.185/175.314	MK906385	MK906480	MK906570	*B
SDEI-Dipt-0001334	*Psl. wernermohrigi*	*macrotegmenta* s. str. clade	SDEI	North Island	Katikati	-37.55/175.90	MK906387	MK906482	MK906572	*B
SDEI-Dipt-0001373	*Psl. wernermohrigi*	*macrotegmenta* s. str. clade	SDEI	North Island	Katikati	-37.55/175.90	MK906394	MK906489	MK906579	*B
SDEI-Dipt-0001386	*Psl. whakahara*	*jejuna* clade	SDEI	South Island	Kahurangi National Park	-41.096/172.704	OQ644728	OQ641724	-	
SDEI-Dipt-0001439	*Psl. whakahara*	*jejuna* clade	NZAC (NZAC02029048)	South Island	Kahurangi National Park	-41.096/172.704	MK906408	MK906492	MK906582	*B
SDEI-Dipt-0001336	*Psl. whena*	*jejuna* clade	NZAC (NZAC02029049)	North Island	Katikati	-37.55/175.90	MK906388	MK906483.2	MK906573	*B
SDEI-Dipt-0000622	*Psl. zealandica*	*zealandica* clade	NZAC (NZAC02028996)	North Island	Tongariro National Park	-39.393/175.450	OQ569819	OQ581681	-	*B/H
SDEI-Dipt-0000623	*Psl. zealandica*	*zealandica* clade	SDEI	North Island	Tongariro National Park	-39.393/175.450	MK906328	MK906419	MK906509	H
SDEI-Dipt-0000783	*Psl. zealandica*	*zealandica* clade	SDEI	North Island	Tongariro National Park	-39.376/175.444	OQ569824	OQ581684	-	*B/H
SDEI-Dipt-0000882	*Psl. zealandica*	*zealandica* clade	SDEI	South Island	Fiordland National Park	-44.699/168.108	OQ569834	OQ581692	-	H
SDEI-Dipt-0000883	*Psl. zealandica*	*zealandica* clade	NZAC (NZAC02029008)	South Island	Fiordland National Park	-44.699/168.108	OQ569835	OQ581693	-	H
SDEI-Dipt-0000884	*Psl. zealandica*	*zealandica* clade	SDEI	South Island	Fiordland National Park	-44.699/168.108	OQ569836	-	-	H

Specimen number	Species	Taxonomic assignment	Voucher deposition	Island	Locality	Coordinates (lat./long.)	GenBank accession numbers			
							COI	16S	28S	
SDEI-Dipt-0000886	*Psl. zealandica*	*zealandica* clade	SDEI	South Island	Fiordland National Park	-44.699/168.108	MK906349	MK906441	MK906531.2	*B/H
SDEI-Dipt-0000998	*Psl. zealandica*	*zealandica* clade	NZAC (NZAC02028994)	North Island	Tongariro National Park	-39.393/175.450	MK906360	MK906452	MK906542	*B/H
SDEI-Dipt-0001001	*Psl. zealandica*	*zealandica* clade	SDEI	North Island	Tongariro National Park	-39.393/175.450	OQ569854	OQ581709	-	H
SDEI-Dipt-0001002	*Psl. zealandica*	*zealandica* clade	NZAC (NZAC02028995)	North Island	Tongariro National Park	-39.393/175.450	OQ569855	OQ581710	-	*B/H
SDEI-Dipt-0001162	*Psl. zealandica*	*zealandica* clade	SDEI	South Island	Cass	-43.033/171.75	OQ573597	OQ598583	-	*B/H
SDEI-Dipt-0001163	*Psl. zealandica*	*zealandica* clade	SDEI	South Island	Cass	-43.033/171.75	OQ573598	OQ598584	-	*B/H
SDEI-Dipt-0001182	*Psl. zealandica*	*zealandica* clade	SDEI	South Island	Cass	-43.033/171.75	OQ573608	OQ598594	-	*B/H
SDEI-Dipt-0001239	*Psl. zealandica*	*zealandica* clade	SDEI	North Island	Tongariro National Park	-39.393/175.450	OQ573611	OQ598597	-	H
SDEI-Dipt-0001242	*Psl. zealandica*	*zealandica* clade	SDEI	North Island	Tongariro National Park	-39.393/175.450	OQ573612	OQ598598	-	H
SDEI-Dipt-0001243	*Psl. zealandica*	*zealandica* clade	NZAC (NZAC02029010)	North Island	Tongariro National Park	-39.393/175.450	OQ573613	OQ598599	-	H
SDEI-Dipt-0001244	*Psl. zealandica*	*zealandica* clade	SDEI	North Island	Tongariro National Park	-39.393/175.450	OQ573614	OQ598600	-	H
SDEI-Dipt-0001246	*Psl. zealandica*	*zealandica* clade	NZAC (NZAC02029009)	North Island	Tongariro National Park	-39.393/175.450	OQ573615	OQ598601	-	*B/H
SDEI-Dipt-0001248	*Psl. zealandica*	*zealandica* clade	SDEI	North Island	Tongariro National Park	-39.393/175.450	OQ573616	OQ598602	-	H
SDEI-Dipt-0001254	*Psl. zealandica*	*zealandica* clade	SDEI	North Island	Tongariro National Park	-39.393/175.450	OQ573617	OQ598603	-	H
SDEI-Dipt-0001538	*Psl. zealandica*	*zealandica* clade	SDEI	North Island	Tongariro National Park	-39.393/175.450	OQ573634	OQ598619	-	H
SDEI-Dipt-0001539	*Psl. zealandica*	*zealandica* clade	SDEI	North Island	Tongariro National Park	-39.393/175.450	OQ573635	OQ598620	-	H
SDEI-Dipt-0001540	*Psl. zealandica*	*zealandica* clade	SDEI	North Island	Tongariro National Park	-39.393/175.450	OQ573636	OQ598621	-	H

Specimen number	Species	Taxonomic assignment	Voucher deposition	Island	Locality	Coordinates (lat./long.)	GenBank accession numbers			
							COI	16S	28S	
SDEI-Dipt-0001541	*Psl. zealandica*	*zealandica* clade	SDEI	North Island	Tongariro National Park	-39.393/175.450	OQ573637	OQ598622	-	H
SDEI-Dipt-0001542	*Psl. zealandica*	*zealandica* clade	SDEI	North Island	Tongariro National Park	-39.393/175.450	OQ573638	OQ598623	-	H
SDEI-Dipt-0001543	*Psl. zealandica*	*zealandica* clade	SDEI	South Island	Fiordland National Park	-44.699/168.108	OQ573639	OQ598624	-	H
SDEI-Dipt-0001544	*Psl. zealandica*	*zealandica* clade	NZAC (NZAC02029007)	South Island	Fiordland National Park	-44.699/168.108	OQ573640	OQ598625	-	H
SDEI-Dipt-0001545	*Psl. zealandica*	*zealandica* clade	SDEI	South Island	Fiordland National Park	-44.699/168.108	OQ573641	OQ598626	-	H
SDEI-Dipt-0001546	*Psl. zealandica*	*zealandica* clade	NZAC (NZAC02029006)	South Island	Fiordland National Park	-44.699/168.108	OQ573642	OQ598627	-	H
SDEI-Dipt-0001547	*Psl. zealandica*	*zealandica* clade	SDEI	South Island	Fiordland National Park	-44.699/168.108	OQ573643	OQ598628	-	H
SDEI-Dipt-0001548	*Psl. zealandica*	*zealandica* clade	NZAC (NZAC02029005)	South Island	Fiordland National Park	-44.699/168.108	OQ573644	OQ598629	-	H
SDEI-Dipt-0001549	*Psl. zealandica*	*zealandica* clade	SDEI	South Island	Fiordland National Park	-44.699/168.108	OQ573645	OQ598630	-	H
SDEI-Dipt-0001550	*Psl. zealandica*	*zealandica* clade	SDEI	South Island	Fiordland National Park	-44.699/168.108	OQ573646	OQ598631	-	H
SDEI-Dipt-0001551	*Psl. zealandica*	*zealandica* clade	NZAC (NZAC02029003)	South Island	Fiordland National Park	-44.699/168.108	OQ573647	OQ598632	-	H
SDEI-Dipt-0001552	*Psl. zealandica*	*zealandica* clade	SDEI	South Island	Fiordland National Park	-44.699/168.108	OQ573648	OQ598633	-	H
SDEI-Dipt-0001553	*Psl. zealandica*	*zealandica* clade	SDEI	South Island	Fiordland National Park	-44.699/168.108	OQ573649	OQ598634	-	*B/H
SDEI-Dipt-0001554	*Psl. zealandica*	*zealandica* clade	NZAC (NZAC02029004)	South Island	Fiordland National Park	-44.699/168.108	OQ573650	OQ598635	-	H
SDEI-Dipt-0001555	*Psl. zealandica*	*zealandica* clade	SDEI	South Island	Fiordland National Park	-44.699/168.108	OQ573651	OQ598636	-	H
SDEI-Dipt-0001556	*Psl. zealandica*	*zealandica* clade	SDEI	South Island	Fiordland National Park	-44.699/168.108	OQ573652	OQ598637	-	H
SDEI-Dipt-0001557	*Psl. zealandica*	*zealandica* clade	NZAC (NZAC02029001)	South Island	Fiordland National Park	-44.699/168.108	OQ573653	OQ598638	-	H

Specimen number	Species	Taxonomic assignment	Voucher deposition	Island	Locality	Coordinates (lat./long.)	GenBank accession numbers			
							COI	16S	28S	
SDEI-Dipt-0001558	*Psl. zealandica*	*zealandica* clade	SDEI	South Island	Fiordland National Park	-44.699/168.108	OQ573654	OQ598639	-	H
SDEI-Dipt-0001559	*Psl. zealandica*	*zealandica* clade	NZAC (NZAC02029002)	South Island	Fiordland National Park	-44.699/168.108	OQ573655	-	-	H
SDEI-Dipt-0001664	*Psl. zealandica*	*zealandica* clade	SDEI	North Island	Tongariro National Park	-39.393/175.450	OQ573674	OQ641748	-	H
SDEI-Dipt-0001669	*Psl. zealandica*	*zealandica* clade	SDEI	North Island	Tongariro National Park	-39.393/175.450	OQ573676	OQ641751	-	H
SDEI-Dipt-0001673	*Psl. zealandica*	*zealandica* clade	SDEI	North Island	Tongariro National Park	-39.393/175.450	OQ573677	OQ641753	-	H

Table S2. Time estimates and probability values for the MRCA distribution for each node of Figure 4. Time estimates are rounded to the nearest of 10 ka. Colours of column headers correspond with the pie charts in Figure 4 (NI, North Island; nSI, northern South Island; sSI, southern South Island; TAS, Tasmania).

				MRCA distribution															
**Node label**	**Taxonomic assignment**	**Mean [Ma]**	**95% HPD-interval [Ma]**	**NI**	**nSI**	**sSI**	**nSI-sSI**	**NI-nSI-TAS**	**nSI-TAS**	**NI-sSI-TAS**	**nSI-sSI-TAS**	**sSI-TAS**	**NI-nSI-sSI-TAS**	**NI-nSI**	**NI-TAS**	**NI-nSI-sSI**	**TAS**	**NI-sSI**	**unclear state**
1	*macrotegmenta* s. l. clade	2.980	1.873–4.301	-	-	-	-	12.2%	11.3%	10.5%	9.9%	9.0%	7.2%	5.9%	5.7%	5.6%	5.2%	5.0%	12.5%
2	*macrotegmenta* s. l. clade	1.967	1.287–2.594	-	-	-	-	15.3%	8.0%	6.0%	-	-	5.3%	-	43.5%	-	17.9%	-	4.0%
3	*macrotegmenta* s. l. clade	1.575	0.479–2.486	-	-	-	-	-	-	-	-	-	-	-	-	-	100%	-	-
4	*macrotegmenta* s. l. clade	1.436	0.384–2.331	-	-	-	-	-	-	-	-	-	-	-	-	-	100%	-	-
5	*macrotegmenta* s. str. clade	0.675	0.430–0.961	63.1%	-	-	-	-	-	-	-	-	-	28.1%	-	6.3%	-	-	2.5%
6	*macrotegmenta* s. str. clade	0.557	0.296–0.827	95.9%	-	-	-	-	-	-	-	-	-	-	-	-	-	-	4.0%
7	*macrotegmenta* s. str. clade	0.014	0–0.036	100%	-	-	-	-	-	-	-	-	-	-	-	-	-	-	-
8	*macrotegmenta* s. str. clade	0.417	0.183–0.653	81.1%	-	-	-	-	-	-	-	-	-	5.1%	-	8.9%	-	-	4.8%
9	*macrotegmenta* s. str. clade	0.259	0.068–0.451	52.6%	-	-	-	-	-	-	-	-	-	15.0%	-	18.2%	-	14.2%	-
10	*macrotegmenta* s. str. clade	0.085	0.014–0.164	37.9%	-	-	-	-	-	-	-	-	-	15.8%	-	33.0%	-	13.3%	-
11	*macrotegmenta* s. str. clade	0.030	0.003–0.069	12.3%	-	-	-	-	-	-	-	-	-	15.5%	-	59.2%	-	12.5%	0.5%
12	*macrotegmenta* s. str. clade	0.012	0–0.034	-	-	-	-	-	-	-	-	-	-	-	-	80.4%	-	17.6%	2.0%
13	*macrotegmenta* s. str. clade	0.028	0–0.081	-	-	-	-	-	-	-	-	-	-	-	-	93.6%	-	-	6.4%
14	*macrotegmenta* s. str. clade	0.363	0.189–0.566	52.3%	-	-	-	-	-	-	-	-	-	47.7%	-	-	-	-	-
15	*macrotegmenta* s. str. clade	0.222	0–0.402	7.0%	-	-	-	-	-	-	-	-	-	93.0%	-	-	-	-	-
16	*macrotegmenta* s. str. clade	0.195	0.027–0.349	100%	-	-	-	-	-	-	-	-	-	-	-	-	-	-	-
17	*macrotegmenta* s. l. clade	0.302	0.040–0.544	-	-	-	97.7%	-	-	-	-	-	-	-	-	-	-	-	2.3%

Table S3. Time estimates and probability values for the MRCA distribution for each node of Figure 5. Time estimates are rounded to the nearest of 10 ka. Colours of column headers correspond with the pie charts in Figure 5 (NI, North Island; nSI, northern South Island; sSI, southern South Island).

				MRCA distribution							
**Node label**	**Taxonomic assignment**	**Mean [Ma]**	**95% HPD-interval [Ma]**	**NI**	**nSI**	**sSI**	**nSI-sSI**	**NI-nSI**	**NI-nSI-sSI**	**NI-sSI**	**unclear state**
18	N. N.	9.945	7.452–12.375	51.8%	-	-	-	30.0%	9.4%	-	8.9%
19	*zealandica* clade	4.950	3.641–6.352	20.4%	-	-	-	72.8%	-	-	6.8%
20	*zealandica* clade	3.722	2.364–5.095	92.9%	-	-	-	-	-	-	7.1%
21	*zealandica* clade	0.352	0.132–0.563	92.8%	-	-	-	-	-	-	7.2%
22	*zealandica* clade	0.111	0.009–0.225	-	-	-	93.7%	-	-	-	6.3%
23	N. N.	8.895	7.127–10.792	67.8%	-	-	-	16.9%	7.8%	-	7.5%
24	*bispina* clade	7.897	6.020–9.622	87.3%	-	-	-	-	-	-	12.7%
25	*bispina* clade	5.630	4.239–6.884	79.8%	-	-	-	10.8%	-	-	9.3%
26	*bispina* clade	4.306	3.033–5.692	71.9%	-	-	-	16.5%	5.4%	-	6.3%
27	*bispina* clade	2.212	1.396–3.023	-	-	-	-	41.0%	49.3%	-	9.7%
28	*bispina* clade	0.558	0.211–0.891	93.9%	-	-	-	-	-	-	6.1%
29	*bispina* clade	0.218	0.026–0.398	93.7%	-	-	-	-	-	-	6.3%
30	*bispina* clade	2.638	1.047–3.830	93.9%	-	-	-	-	-	-	6.1%
31	*bispina* clade	0.541	0.160–0.916	93.8%	-	-	-	-	-	-	6.2%
32	*bispina* clade	1.235	0.515–1.999	-	-	-	-	93.5%	-	-	6.5%
33	*jejuna* clade	6.093	4.918–7.413	29.0%	-	-	-	22.8%	41.9%	-	6.3%
34	*jejuna* clade	4.447	3.280–5.618	93.7%	-	-	-	-	-	-	6.3%
35	*jejuna* clade	2.718	1.992–3.610	93.7%	-	-	-	-	-	-	6.3%
36	*jejuna* clade	1.725	1.017–2.465	85.7%	-	-	-	8.1%	-	-	6.2%
37	*jejuna* clade	5.554	4.401–6.674	15.4%	-	-	16.2%	15.0%	43.0%	-	10.4%
38	*jejuna* clade	4.557	3.480–5.625	-	-	35.6%	17.2%	-	40.7%	-	6.5%
39	*jejuna* clade	3.460	2.453–4.377	-	-	23.9%	18.2%	-	51.8%	-	6.1%
40	*jejuna* clade	1.942	1.298–2.687	-	-	-	-	19.3%	74.6%	-	6.1%
41	*jejuna* clade	0.802	0.357–1.162	-	13.4%	-	80.3%	-	-	-	6.3%

Node label	Taxonomic assignment	Mean [Ma]	95% HPD-interval [Ma]	MRCA distribution							
				NI	nSI	sSI	nSI-sSI	NI-nSI	NI-nSI-sSI	NI-sSI	unclear state
42	*jejuna* clade	0.397	0.112–0.663	-	-	-	94.7%	-	-	-	5.3%
43	*jejuna* clade	2.914	1.629–3.932	14.4%	-	-	-	20.2%	59.3%	-	6.1%
44	*jejuna* clade	1.459	0.669–2.162	-	-	-	5.5%	-	88.2%	-	6.3%
45	*jejuna* clade	0.215	0.075–0.369	-	-	93.7%	-	-	-	-	6.3%
46	*jejuna* clade	0.134	0.003–0.266	-	-	80.0%	11.5%	-	-	-	8.5%
